# Association of Psychological Safety with PTSD Symptoms Among Regional Firefighters in South Korea: Moderating Roles of Occupational Identity and Peer Support

**DOI:** 10.3390/ijerph23050635

**Published:** 2026-05-11

**Authors:** Jea-Yong Jung, Gwi-Gon Kim

**Affiliations:** Department of Business Administration, Kumoh National Institute of Technology, Gumi 39177, Republic of Korea; gh129@korea.kr

**Keywords:** firefighters, occupational health, occupational identity, peer support, psychological safety, PTSD symptoms

## Abstract

**Highlights:**

**Public health relevance—How does this work relate to a public health issue?**

- Firefighters are repeatedly exposed to occupational trauma, making PTSD symptoms a critical public health concern in high-risk occupational contexts.
- Organizational conditions, beyond individual vulnerability, are relevant to understanding and managing occupational mental health risks at the public health level.

**Public health significance—Why is this work of significance to public health?**

- This study examines the association between psychological safety and PTSD symptoms among firefighters within an occupational health and public health framework, contributing to the understanding of occupational mental health in high-risk contexts.
- This study further tests whether occupational identity and peer support condition this association, providing evidence on the role of social and organizational resources in occupational mental health in high-risk occupations.

**Public health implications—What are the key implications or messages for practitioners, policy makers and/or researchers in public health?**

- Psychological safety may be incorporated into organizational strategies aimed at preventing and managing occupational mental health risks in high-risk occupations.
- Strengthening occupational identity and peer support may help identify conditions under which psychological safety is more strongly related to PTSD symptoms, thereby informing workplace-based mental health interventions.

**Abstract:**

Firefighters are repeatedly exposed to occupational trauma in high-risk work settings, and PTSD symptoms represent an important occupational and public health concern. This study examined the association between psychological safety and PTSD symptoms among firefighters and tested whether this association is conditioned by occupational identity and peer support within an occupational health framework. A cross-sectional survey was conducted with 314 firefighters in Gyeongsangbuk-do, South Korea. PTSD symptoms were assessed using eight selected items adapted from the PCL-5 representing core symptom domains. Confirmatory factor analysis, correlation analysis, and regression analysis were performed, and moderation analyses were conducted using PROCESS Macro (Model 1). Results indicated that psychological safety was negatively associated with PTSD symptoms. Both occupational identity and peer support significantly conditioned this association. The negative association was consistently observed across all levels of occupational identity, whereas it was significant only at or above the mean level of peer support. These findings suggest that psychological safety may be understood as an organizational resource related to PTSD symptoms in high-risk occupational contexts, while occupational identity and peer support may function as individual and social resources that condition this association. The findings highlight the importance of understanding occupational mental health based on the integrated roles of organizational, individual, and social resources.

## 1. Introduction

### 1.1. Occupational Trauma Exposure and Mental Health Among Firefighters

Firefighters are classified as a representative high-risk occupational group who are repeatedly exposed to potentially traumatic events during fire suppression, rescue, and emergency response operations [1]. Traumatic experiences in firefighting involve exposure to life-threatening or severely distressing incidents, and PTSD symptoms commonly include intrusion, avoidance, negative cognitive or mood changes, and hyperarousal. A systematic review of disaster response personnel reported that occupational trauma exposure is a major occupational risk factor associated with post-traumatic stress disorder (PTSD) symptoms [2]. Studies on public safety personnel, including military personnel and firefighters, have also indicated that the prevalence of PTSD and its related factors are associated with job characteristics and organizational contexts [3,4]. In addition, meta-analyses of firefighters and rescue personnel have reported that the prevalence of PTSD ranges from 6.4% to 57% [5,6], and variations have been observed depending on differences between professional and volunteer firefighters as well as national contexts [7]. These variations suggest that, in addition to the repeated nature of trauma exposure, organizational conditions and operational contexts within public safety organizations should also be considered [8]. In addition to fatalities, severe injuries, and critical incidents, PTSD risk may also be associated with exposure to violence, aggression, and other potentially threatening interactions encountered during emergency response work.

Previous research has suggested that mental health burdens arising from repeated trauma exposure in structured work environments may not be fully explained by individual psychological vulnerability alone [2]. Rather than being reduced to individual vulnerability, PTSD symptoms may be understood as outcomes related to accumulated occupational risks shaped by repeated exposure and organizational contexts [6,8]. This perspective indicates that PTSD symptoms are not solely the result of single events but are associated with occupational risk structures in which cumulative exposure and organizational conditions are jointly involved. In addition, mental health outcomes in public safety organizations need to be understood within the context of psychosocial risk management, including job demands, recovery opportunities, and organizational support [9,10].

According to national fire service statistics in South Korea, in 2024 there were 3,324,287 emergency medical responses, 1,318,837 rescue operations, and 37,614 fire responses, with 2 line-of-duty deaths and 1445 injuries reported [11]. These figures indicate that firefighting work involves frequent exposure to physical risks and suggest that psychosocial risks may also accumulate alongside repeated incident responses. A nationwide survey in South Korea reported PTSD symptoms among firefighters and barriers to treatment utilization [12], and the prevalence of suicidal ideation and related factors has also been documented [13]. Prior studies suggest that sociodemographic characteristics, including age, tenure, rank, and marital status, may be associated with PTSD symptom patterns among firefighters [6,13]. Furthermore, a longitudinal study of newly recruited firefighters showed that repeated trauma exposure during early career stages was associated with PTSD and depressive symptoms [14]. Prior studies suggest that repeated critical incidents, cumulative trauma exposure, high emotional demands, and unpredictable emergency operations are associated with PTSD risk among firefighters. These findings support the view that structured work environments characterized by repeated exposure may be consistently associated with mental health indicators.

PTSD symptoms have been reported to be associated with functional impairment, mental health comorbidities, and health-related quality of life [15,16,17]. Safety climate and emotional exhaustion have also been linked to job performance and well-being indicators [18]. Safety climate and leadership have been reported to be associated with safety behaviors [19,20], and managerial support has been discussed as a factor related to the mental health of emergency responders [21]. These findings indicate that mental health issues among firefighters are not limited to individual suffering but may also be related to organizational functioning, safety performance, workforce retention, and the sustainability of public safety systems.

PTSD symptoms related to repeated trauma exposure may be addressed within prevention-oriented occupational health management systems rather than being limited to treatment-focused approaches, and discussions have emphasized organizational-level implementation and practical application [22,23]. In high-risk occupational contexts where repeated exposure persists, it is important to understand how psychosocial resources that can be managed at the organizational level are related to mental health indicators [9,24]. In this context, there is a need for empirical analysis of how organizational resources are associated with PTSD symptoms and under what conditions such associations become more evident [8,24]. This perspective also suggests that occupational identity may shape whether psychological safety is recognized as a meaningful occupational resource, supporting its conceptualization as a moderator.

Previous studies have primarily focused on identifying the association between trauma exposure and PTSD symptoms [6,7,13], and some studies have examined organizational factors or social resources separately [24]. However, studies that simultaneously consider organizational, individual, and social resources in high-risk occupational contexts and examine the conditions under which these resources are more strongly associated with mental health indicators remain limited. Research introducing psychological safety as a measurable organizational characteristic has reported that psychological safety may be associated with mental health and job performance indicators [25]. In particular, psychological safety, occupational identity, and peer support can be conceptualized as organizationally and socially modifiable resources. Occupational identity and peer support were conceptualized as moderators because they may condition how psychological safety is interpreted and translated into coping-relevant resources, influencing the strength of its association with PTSD symptoms, rather than functioning solely as parallel predictors or mediating mechanisms. However, empirical studies that simultaneously examine the conditional association of these three factors with PTSD symptoms are still limited. This gap highlights the need to expand the evidence base for developing prevention-oriented occupational health strategies in high-risk occupational settings.

### 1.2. Association Between Psychological Safety and PTSD Symptoms

In high-risk occupational environments characterized by repeated trauma exposure, PTSD symptoms have been reported as a major psychosocial occupational risk [26,27]. Meta-analyses and studies conducted in South Korea have indicated that repeated trauma exposure is associated not only with PTSD symptoms but also with burnout, depression, and suicidal ideation [28,29]. These findings suggest that PTSD should not be reduced to an issue of individual vulnerability but rather understood as an indicator of accumulated occupational risk resulting from repeated exposure in structured work environments [30,31]. From an occupational health perspective, this implies that psychosocial risks, along with physical hazards, should be considered an important domain in occupational safety and health management [32].

Conservation of resources theory suggests that stress-related responses may occur when individuals’ resources are threatened or lost, and that such responses may accumulate when resource loss is repeated [33,34]. Within this framework, work environments characterized by repeated trauma exposure may be associated with continuous depletion of emotional and social resources, and the accumulation of such resource loss may be related to mental health indicators such as PTSD symptoms [35,36]. Organizational and social resources may also function as contextual factors related to the process of resource loss, and it has been reported that the association with PTSD symptoms may vary depending on the level of resources available, even under similar exposure conditions [37].

Psychological safety is defined as an organizational climate in which members do not fear negative consequences when expressing opinions, reporting errors, or seeking help [38]. Subsequent studies have reported that such an organizational climate may be associated with both work environments and mental health outcomes [39]. In risk-sensitive occupations, information sharing and speaking up are considered key elements for ensuring safety [19,40], and such organizational conditions may be related to how psychological burdens arising during task performance are shared within the organization [41,42]. In particular, in contexts of repeated trauma exposure, organizational conditions that allow members to express emotional responses and seek support may be associated with the mitigation of resource loss [43]. Recent studies have reported that psychological safety is associated with mental health indicators and job attitudes [25], and systematic reviews have shown that this construct is increasingly considered an important organizational resource in the field of occupational health [44].

Taken together, psychological safety does not eliminate exposure to trauma itself but may be understood as an organizational resource related to an environment in which members can report emotional responses and seek support [25,39]. In this sense, psychological safety may be relevant not only as a general organizational climate but also as a resource context associated with how occupational stressors are interpreted and managed. However, studies that directly examine the association between psychological safety and PTSD symptoms in structured firefighting environments characterized by repeated trauma exposure remain limited. In particular, the ways in which organizational contexts—such as reporting and help-seeking practices following traumatic experiences and mutual trust within the organization—are associated with PTSD symptoms have not been sufficiently clarified [41].

Accordingly, this study conceptualizes psychological safety as a modifiable organizational resource that may be related to lower PTSD symptom levels under repeated trauma exposure and examines this association among firefighters. Based on this perspective, higher psychological safety was expected to be negatively associated with PTSD symptom levels.

**H1.**  *Higher psychological safety will be negatively associated with PTSD symptoms among firefighters.*

### 1.3. Moderating Role of Occupational Identity

Occupational identity is defined as an identity-based resource through which individuals integrate their occupation into their self-concept and assign meaning to their work experiences [45,46]. In high-risk occupational environments, occupational identity has been reported to function as a frame of reference for interpreting and meaning-making of work experiences and emotional demands [47]. This identity may also be related to how individuals perceive and respond to emotional demands encountered during job performance [48]. Following trauma exposure, mental health indicators, including PTSD symptoms, may present differently across individuals, and such variations have been suggested to be related to identity-based resources possessed by individuals [49,50].

Previous studies have reported that occupational identity may be related to job stress, burnout, and other mental health indicators [51,52]. In addition, occupational identity has been associated with job satisfaction and psychological well-being and may be understood as an individual resource related to the process of assigning meaning to work experiences [53]. These findings suggest that occupational identity may function as an individual resource through which work experiences are interpreted and meaningfully organized.

According to conservation of resources theory, stress-related outcomes may be conditioned by the interaction between individual and environmental resources [54,55]. In firefighting environments characterized by repeated trauma exposure, PTSD symptoms may be understood as mental health indicators related to accumulated occupational risk [56,57]. Within this context, occupational identity may be conceptualized as a form of individual resource that shapes how organizational resources are interpreted and utilized. Empirical findings have also suggested that occupational identity may be related to psychological outcomes such as job stress and emotional burden [58,59].

Within this perspective, even when the same organizational environment is provided, the way it is perceived and utilized may vary depending on the level of occupational identity [60,61]. In particular, higher levels of occupational identity may be associated with stronger meaning attribution to work and organization, which may strengthen the extent to which psychological safety is recognized and mobilized as a meaningful organizational resource [62]. Conversely, lower levels of occupational identity may be associated with more limited recognition and utilization of the same organizational resource [63]. These perspectives suggest that the association between organizational resources and mental health indicators may vary depending on the level of individual identity resources. Prior research has also reported that occupational identity moderates the association between person–environment fit and job burnout [64], and that the interaction between identity verification and organizational context may be related to psychological distress [65].

Taken together, occupational identity may be understood as an individual resource that conditions both the perception of organizational resources and the strength of the association between psychological safety and PTSD symptoms. However, studies directly examining whether occupational identity conditions the association between psychological safety and PTSD symptoms among firefighters remain limited [66]. Therefore, this study aims to examine whether occupational identity moderates the association between psychological safety and PTSD symptoms.

**H2.**  *Occupational identity will moderate the negative association between psychological safety and PTSD symptoms.*

### 1.4. Moderating Role of Peer Support

Peer support refers to the exchange of emotional, informational, and instrumental support among colleagues who share similar job demands and trauma experiences [67]. As a form of social support developed within organizational contexts, peer support has been discussed as a social resource related to individuals’ psychological well-being and work experiences under demanding job conditions [68,69]. Support among colleagues has also been reported to be related to job satisfaction and organizational experiences [70]. In particular, in environments characterized by repeated trauma exposure, peer support has been identified as a social resource associated with a sense of belonging and mutual trust [71], and in high-risk occupations, such support has been considered a critical psychosocial resource. Workplace social support has been reported as a resource related to mental health under job stress conditions [72,73], and studies on public safety personnel have discussed peer support programs as organizationally embedded resources associated with experience sharing and emotional support following traumatic events [74,75]. Such environments of mutual support may also be related to experience sharing among members and perceptions of psychological safety within the organization.

According to conservation of resources theory, social support may be understood as a resource related to the process of resource loss [34]. In occupational settings characterized by repeated trauma exposure, PTSD symptoms may be understood as mental health indicators related to resource depletion [56,57]. Within this context, peer support has been reported to be associated with mental health responses following trauma exposure [76]. Social support has also been suggested to be related to emotional responses and coping processes [77]. Furthermore, environments characterized by mutual peer support have been reported to be associated with perceptions of psychological safety within organizational contexts [78].

Studies on firefighters and public safety personnel have reported that peer and organizational social support may be associated with PTSD symptom levels [79,80]. It has also been suggested that the association between organizational environments and mental health indicators may vary depending on the level of trust and accessibility within peer networks [24,81]. This suggests that peer support may shape the extent to which psychological safety is translated into accessible interpersonal coping resources. Empirical studies have further reported that peer support may show a moderating pattern in the association between job stress and mental health indicators [82,83]. These findings suggest that peer support may function as a social resource that conditions the strength of the association between psychological safety and PTSD symptoms.

Taken together, peer support may be understood as a social resource that conditions not only access to interpersonal support but also the strength of the association between psychological safety and PTSD symptoms in high-risk occupational contexts. For example, research on firefighters has reported that the pattern of association with PTSD symptoms may vary depending on the level of peer support [79].

Accordingly, this study aims to examine whether peer support moderates the association between psychological safety and PTSD symptoms.

**H3.**  *Peer support will moderate the negative association between psychological safety and PTSD symptoms.*

### 1.5. Research Objectives

This study aims to (1) examine the association between psychological safety and PTSD symptoms among firefighters and (2) analyze how occupational identity and peer support condition this association. Within high-risk occupational environments characterized by structured and repeated trauma exposure, this study seeks to empirically examine, from an occupational health perspective, the conditional association between modifiable organizational and peer-based resources and mental health indicators.

**Figure 1:**
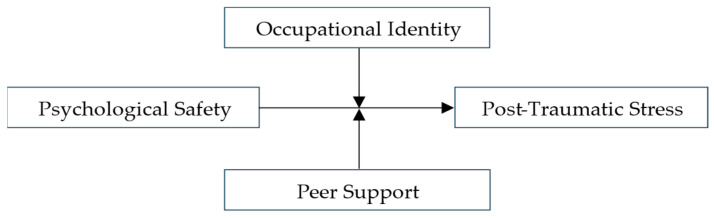
Conceptual model illustrating the association between psychological safety and PTSD symptoms, moderated by occupational identity and peer support.

This approach extends the understanding of PTSD symptoms beyond individual vulnerability by situating them within modifiable organizational and social resource contexts relevant to occupational health practice. The conceptual framework of the study is presented in Figure 1.

## 2. Materials and Methods

### 2.1. Study Design and Participants

This study employed a cross-sectional survey design to examine the association between psychological safety and PTSD symptoms among firefighters and to investigate how this association is conditioned by occupational identity and peer support. Rather than inferring causal effects, the study focused on empirically examining the associations and conditional patterns among theoretically aligned variables within an occupational health framework.

This study was conducted with approval from the Institutional Review Board of Kumoh National Institute of Technology (IRB No. 202508-HR-001-01, approved on 19 August 2025) and adhered to the ethical standards of the Declaration of Helsinki. Data were collected over approximately two weeks, from 26 August to 8 September 2025, using an anonymous online self-report survey. The survey was distributed through official organizational communication channels to active-duty firefighters working in fire stations and 119 safety centers within a regional fire service organization in South Korea.

Participation was voluntary. Prior to the survey, participants were provided with information regarding the purpose of the study, data collection procedures, anonymity, data protection measures, and their right to withdraw at any time. All participants provided informed consent electronically before completing the survey. No personally identifiable information was collected. Survey responses were not accessible to supervisors or organizational administrators.

A total of 317 responses were obtained. After screening for abnormal response patterns and standardized residuals exceeding ±3, three cases were excluded as outliers, resulting in a final analytic sample of 314 participants. The mean age of participants was 42.7 years, the mean tenure was 12.4 years, and 89.5% were male. These findings are best understood within the context of a regional firefighter sample rather than as nationally representative of all South Korean firefighters.

Cross-sectional designs are widely used to examine associations between job-related factors and health indicators [84,85], although they have limitations in establishing temporal order [86]. Accordingly, this study avoided causal interpretation and focused on associations and conditional patterns among variables [85,87].

### 2.2. Measures

All variables were measured using a 7-point Likert scale ranging from 1 (strongly disagree) to 7 (strongly agree). The measures were based on previously validated scales with established reliability and validity, with some items modified to reflect the firefighting work context. All items were translated into Korean and finalized through a review process among the research team to ensure clarity and contextual appropriateness.

Psychological safety refers to the extent to which individuals perceive that they can engage in interpersonal risk-taking behaviors, such as expressing opinions, asking questions, and reporting mistakes, without fear of negative consequences [38,88]. Five items were adopted based on Edmondson’s scale. Based on confirmatory factor analysis, one item with a standardized factor loading below 0.60 was excluded, and four items were retained for the final analysis. The removed item (PS1) showed insufficient loading on the latent construct, and its exclusion improved measurement adequacy without materially altering the conceptual coverage of psychological safety.

PTSD symptoms were measured using the PTSD Checklist for DSM-5 (PCL-5) [89]. In this study, eight items representing the core DSM-5 symptom domains were selected to reflect the context of repeated occupational trauma exposure. This approach was intended to retain the core construct while minimizing respondent burden. As a self-report symptom assessment tool, the abbreviated PCL-5 measure was used to assess trauma-related stress responses and capture core occupationally relevant PTSD symptom domains rather than replicate the full diagnostic instrument or serve as a clinical diagnostic tool.

Occupational identity refers to the extent to which individuals integrate their occupation into their self-concept and experience a sense of belonging and pride through their work [90,91]. It was measured using six items adapted from the scales developed by Hall and Snizek.

Peer support refers to the perceived level of emotional, informational, and instrumental support provided by colleagues within the organization [92]. Based on the conceptualization of Caplan et al., six items were measured with reference to prior studies on public safety personnel [79,93].

### 2.3. Data Analysis

To examine the association between psychological safety and PTSD symptoms and to investigate how this association is conditioned by occupational identity and peer support, a structured analytical procedure was applied. Descriptive statistics, reliability analysis, and regression analyses were conducted using IBM SPSS Statistics version 23.0 (IBM Corp., Armonk, NY, USA). Confirmatory factor analysis was performed using IBM SPSS AMOS Graphics version 23.0 (IBM Corp., Armonk, NY, USA). First, confirmatory factor analysis (CFA) was conducted using maximum likelihood estimation to assess the fit of the measurement model as well as the reliability and validity of the constructs [94]. Maximum likelihood estimation was considered appropriate because the indicators were measured on 7-point scales approximating continuous variables, and preliminary screening did not indicate severe departures from multivariate normality.

Model fit was evaluated using the comparative fit index (CFI), Tucker–Lewis index (TLI), root mean square error of approximation (RMSEA), and standardized root mean square residual (SRMR) [95,96]. Internal consistency was assessed using Cronbach’s α and McDonald’s ω. Convergent validity was evaluated based on standardized factor loadings (≥0.50) and average variance extracted (AVE ≥ 0.50) [97]. Composite reliability and AVE were calculated using the lavaan and semTools packages in R version 4.5.2 (R Foundation for Statistical Computing, Vienna, Austria) [98].

Next, descriptive statistics (means and standard deviations) and Pearson correlation analyses were conducted for the main variables. To examine whether the association between psychological safety and PTSD symptoms is conditioned across levels of occupational identity and peer support, moderation analyses were performed using PROCESS Macro version 4.2 for SPSS (Model 1; Andrew F. Hayes, Columbus, OH, USA) [99]. All continuous variables were mean-centered, and 95% confidence intervals were estimated using 5000 bootstrap samples. When interaction terms were significant, simple slope analyses and the Johnson–Neyman technique were applied to interpret conditional association patterns across levels of the moderators. Position (managerial = 1, non-managerial = 0) and tenure (≥10 years = 1, <10 years = 0) were included as control variables. These controls were selected to account for structural variation in occupational exposure and role responsibility that may be relevant to PTSD symptom levels in high-risk work contexts [9,24]. Position was dichotomized to distinguish supervisory and non-supervisory roles consistent with this rationale. Age was not additionally included because of conceptual overlap with tenure, and sex was not prioritized because of limited variability in the sample. Diagnostic checks indicated no serious concerns regarding multicollinearity, influential observations, linearity, heteroscedasticity, or residual assumptions.

Finally, the potential for common method variance (CMV) due to the use of self-report data was assessed [100]. As procedural remedies, anonymity of responses was ensured and item order was randomized. For statistical assessment, Harman’s single-factor test and a single-factor CFA model were applied, and their fit was compared with that of the proposed measurement model [101,102,103].

## 3. Results

### 3.1. Demographic Characteristics of Participants

Table 1 presents the demographic characteristics of the 314 participants. The majority of participants were male (89.5%). In terms of age distribution, participants in their 30s (33.4%), 40s (29.6%), and 50 years or older (34.7%) accounted for the largest proportions.

Regarding marital status, 85.0% of participants were married. In terms of education level, the majority were college graduates (76.1%).

With respect to position, fire lieutenants (26.8%), senior firefighters (25.2%), and captains or higher ranks (23.6%) comprised the largest groups.

In terms of years of service, participants with 10–20 years (28.0%) and more than 20 years (36.6%) of service represented the majority of the sample.

### 3.2. Measurement Model and Validity Assessment

To validate the measurement model consisting of four constructs—psychological safety, post-traumatic stress (PTSD) symptoms, occupational identity, and peer support—confirmatory factor analysis (CFA) was conducted.

The results indicated that the measurement model demonstrated an acceptable level of fit: χ^2^(246) = 759.718, *p* < 0.001, CFI = 0.915, TLI = 0.905, RMSEA = 0.082 (90% CI [0.075–0.088]), and SRMR = 0.048. These values are generally considered acceptable according to commonly suggested model fit criteria [96] (Table 2).

Standardized factor loadings ranged from 0.611 to 0.893 and were all statistically significant (*p* < 0.001). One item of psychological safety (PS1), which showed a relatively low factor loading in the initial CFA, was excluded from the final measurement model. The standardized factor loadings for all items are presented in Supplementary Table S1.

Regarding reliability and convergent validity, Cronbach’s α ranged from 0.887 to 0.938, and McDonald’s ω ranged from 0.889 to 0.941. The average variance extracted (AVE) ranged from 0.574 to 0.729, exceeding the recommended threshold of 0.50 for all constructs.

Discriminant validity was also supported, as the square roots of AVE for each construct were greater than the inter-construct correlations [97] (Table 3).

### 3.3. Descriptive Statistics and Correlation Analysis

Table 4 presents the means, standard deviations, and correlations among the main study variables. The mean of psychological safety was 26.10 (SD = 6.45), and the mean of PTSD symptoms was 22.97 (SD = 11.54). The means of occupational identity and peer support were 28.32 (SD = 6.69) and 32.86 (SD = 6.79), respectively.

The results of the correlation analysis indicated that psychological safety was significantly negatively related to PTSD symptoms (r = −0.395, *p* < 0.01) and positively related to occupational identity (r = 0.393, *p* < 0.01) and peer support (r = 0.639, *p* < 0.01). PTSD symptoms were significantly negatively related to occupational identity (r = −0.343, *p* < 0.01) and peer support (r = −0.458, *p* < 0.01). In addition, occupational identity was positively related to peer support (r = 0.572, *p* < 0.01).

Bivariate correlations ranged in absolute value from 0.343 to 0.639 and did not suggest substantial overlap among the study variables. Potential multicollinearity was further assessed in the regression analyses using variance inflation factors (VIF), which did not indicate problematic multicollinearity.

### 3.4. Association Between Psychological Safety and PTSD Symptoms (H1)

To examine the association between psychological safety and PTSD symptoms, a regression analysis was conducted including position and tenure as control variables (Table 5).

The results indicated that psychological safety was significantly negatively related to PTSD symptoms (B = −0.707, SE = 0.093, β = −0.395, t = −7.599, *p* < 0.001).

The regression model was statistically significant (F(3, 310) = 19.133, *p* < 0.001) and explained 15.6% of the variance in PTSD symptoms (R^2^ = 0.156). In contrast, position and tenure, which were included as control variables, were not significantly related to PTSD symptoms.

### 3.5. Moderation Analysis

#### 3.5.1. Occupational Identity as a Moderator

To examine whether occupational identity conditions the association between psychological safety and PTSD symptoms, PROCESS Macro (Model 1) was applied (Table 6).

The results indicated that both psychological safety and occupational identity were significantly negatively related to PTSD symptoms. In addition, the interaction term between psychological safety and occupational identity was statistically significant (B = −0.109, SE = 0.050, *p* = 0.028), indicating that occupational identity conditions the association between psychological safety and PTSD symptoms. The model explained 21.2% of the variance in PTSD symptoms (R^2^ = 0.212).

**Figure 2:**
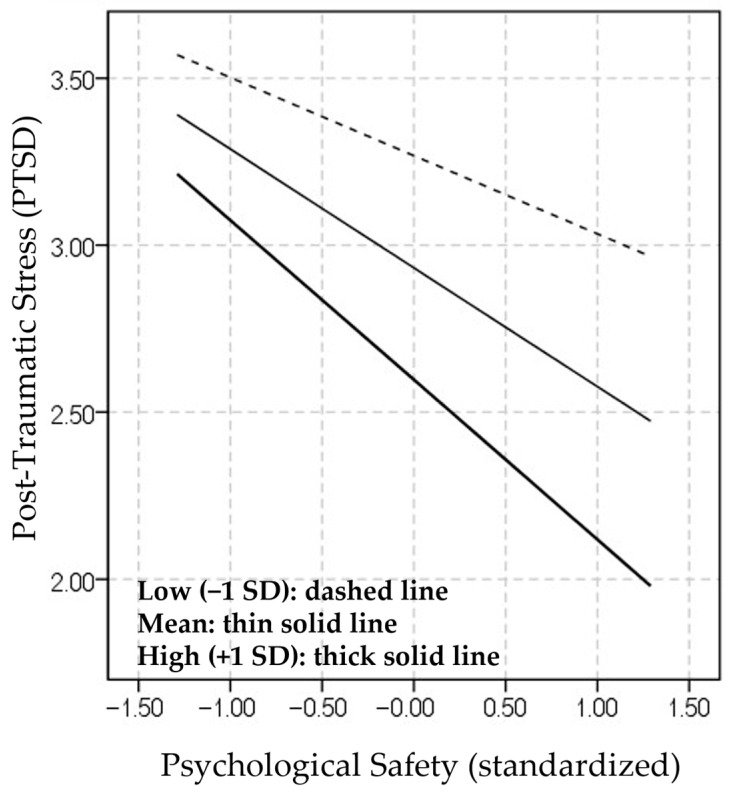
Interaction between psychological safety and occupational identity on PTSD symptoms.

The results of the simple slope analysis showed that the negative association between psychological safety and PTSD symptoms was significant at low (−1 SD), mean, and high (+1 SD) levels of occupational identity (Supplementary Table S2). The interaction pattern is illustrated in Figure 2.

The Johnson–Neyman analysis indicated that the association between psychological safety and PTSD symptoms was statistically significant when occupational identity exceeded −1.553 standard deviations from the mean (Supplementary Figure S1).

#### 3.5.2. Peer Support as a Moderator

To examine whether peer support conditions the association between psychological safety and PTSD symptoms, the same analytical procedure was applied (Table 6).

The results indicated that both psychological safety and peer support were significantly negatively related to PTSD symptoms. The interaction term between psychological safety and peer support was also statistically significant (B = −0.106, SE = 0.047, *p* = 0.023), indicating that peer support conditions the association between psychological safety and PTSD symptoms. The model explained 24.2% of the variance in PTSD symptoms (R^2^ = 0.242).

**Figure 3:**
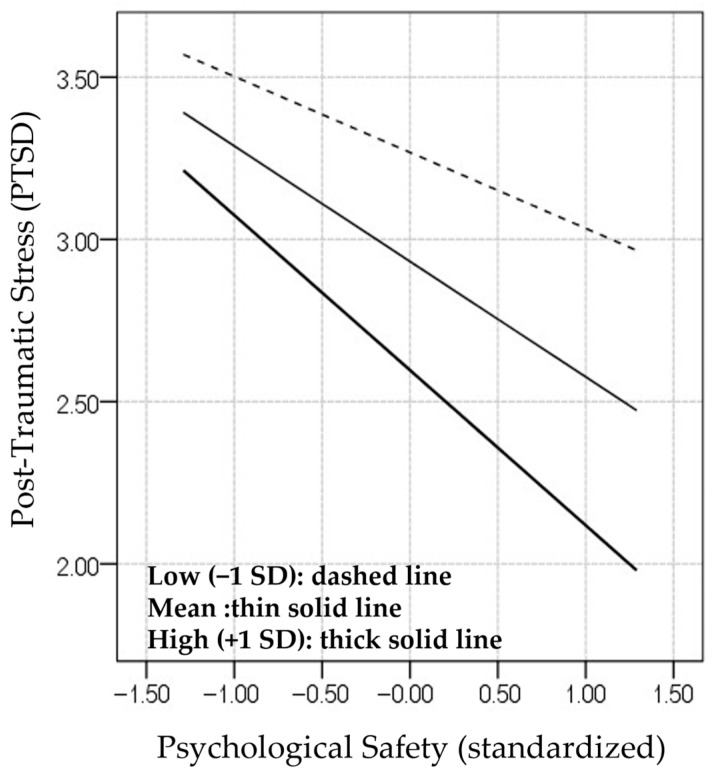
Interaction between psychological safety and peer support on PTSD symptoms.

The results of the simple slope analysis showed that the association between psychological safety and PTSD symptoms was not statistically significant at low levels of peer support (−1 SD), but was significant at mean and high (+1 SD) levels (Supplementary Table S3). The interaction pattern is presented in Figure 3.

The Johnson–Neyman analysis indicated that the association between psychological safety and PTSD symptoms was statistically significant when peer support exceeded −0.504 standard deviations from the mean (Supplementary Table S4; Supplementary Figure S2).

### 3.6. Multicollinearity Diagnostics

To assess multicollinearity, variance inflation factor (VIF) values were examined for the regression models. The results showed that all VIF values ranged from 1.002 to 1.204. As VIF values below 5 are generally considered indicative of no serious multicollinearity concerns [98], these results did not indicate problematic multicollinearity in the present study.

## 4. Discussion

### 4.1. Summary of Findings

This study examined the association between psychological safety and post-traumatic stress (PTSD) symptoms among firefighters in a regional fire service organization in South Korea and investigated how this association is conditioned by occupational identity and peer support.

Under a cross-sectional design controlling for position and tenure, the results of the regression and moderation analyses indicated that psychological safety was negatively related to PTSD symptoms, suggesting that it may be understood as an organizational condition associated with mental health in high-risk occupational contexts. Given the cross-sectional design, however, these findings should be interpreted as associations rather than directional relationships, and alternative explanations, including reverse or reciprocal associations, cannot be ruled out. This finding is consistent with prior research on first responders indicating that trauma-related mental health outcomes are related not only to discrete events but also to repeated exposure and organizational context [66,104].

The findings further indicated that the association between psychological safety and PTSD symptoms is conditioned differently by occupational identity and peer support. Occupational identity showed a pattern in which the negative association between psychological safety and PTSD symptoms was consistently observed across all levels. This pattern suggests that occupational identity may condition the strength, rather than the direction, of the association between psychological safety and PTSD symptoms. In this sense, occupational identity may function as a boundary condition under which this association is more pronounced, consistent with prior research linking occupational identity to emotional work experiences and job involvement in firefighting contexts [48,105].

In contrast, peer support showed a conditional pattern in which the negative association between psychological safety and PTSD symptoms was statistically significant only at average and higher levels. This pattern suggests that peer support may function as a social resource under conditions in which the association between psychological safety and PTSD symptoms is more pronounced [75,106]. This is consistent with prior studies reporting that social support and a sense of belonging among firefighters are negatively related to PTSD symptoms [67,106], and with research indicating that informal peer support may function as a relevant social resource in coping processes following critical incidents [75,93].

Although this study focused on firefighters in South Korea, the issue of repeated trauma exposure and organizationally embedded resources is relevant across public safety personnel. From an occupational health perspective, these findings suggest the importance of understanding mental health not solely at the individual level but within organizational and social contexts.

### 4.2. Theoretical Implications

The findings of this study suggest that, in high-risk occupational contexts characterized by repeated trauma exposure, PTSD symptoms may be understood not solely in terms of individual vulnerability but within an occupational context in which cumulative trauma exposure and organizational, individual, and social resources are jointly related [66,104,107]. Recent discussions have similarly emphasized that the psychological strain of frontline workers is better understood not only within a single-event PTSD framework but as part of accumulated occupational risk shaped by repeated exposure, organizational pressure, and institutional conditions [107].

Psychological safety refers to a shared perception that individuals can express concerns, mistakes, or emotions without fear of negative evaluation or punishment [38]. In this context, such an environment may be related to how emotional tension and psychological burden following trauma exposure are experienced and expressed. Recent reviews in high-risk occupations have identified inclusive leadership, organizational structure, and communication climate as key antecedents of psychological safety [108]. From an occupational health perspective, psychological safety may therefore be understood as an organizational resource related to the expression of difficulties and the seeking of support following work-related traumatic experiences.

In addition, this study highlights a multi-layered resource structure in which the association between psychological safety and PTSD symptoms is conditioned by occupational identity and peer support. Occupational identity and peer support may be considered individual and social resources, respectively, that are related to organizational resources. The interrelation among these resources may be understood within the context of mental health in high-risk occupations. Prior research on firefighters and other first responders has suggested that trauma-related mental health is associated with combinations of factors such as organizational climate, social support, and emotional work experiences rather than a single resource [48,66,75]. The present study extends this perspective by specifying a structured combination of psychological safety, occupational identity, and peer support.

These findings are also consistent with the Job Demands–Resources (JD–R) perspective, which describes how job demands and resources are jointly related to employee outcomes [109,110], and with the Conservation of Resources (COR) perspective, which emphasizes the interrelated and accumulative nature of resources [111]. In particular, the results suggest that organizational, social, and individual resources may operate in conjunction, providing a basis for understanding resource structures in high-risk occupational settings. This interpretation is aligned with prior findings indicating that psychological strain among firefighters is related not only to trauma exposure itself but also to operational climate, social support, and coping-related factors [66].

### 4.3. Practical Implications

The findings of this study suggest that modifiable organizational and social resources may be relevant considerations for understanding occupational mental health in high-risk occupational settings.

First, fire service organizations may consider reviewing management approaches that rely primarily on punitive or error-avoidance practices and instead examine organizational climates characterized by psychological safety. Environments in which personnel perceive that they can raise questions, express concerns, and communicate emotional experiences without fear of negative evaluation may be related to help-seeking and support-seeking behaviors following traumatic exposure [38,112]. Recent research on first responder organizations indicates that workplace mental health programs are discussed not only in terms of individual-level training but also in relation to trust-based communication, stigma reduction, and organizational culture [113]. Participatory prevention programs in fire service contexts have similarly been reported to be related to changes in organizational climate and emotional awareness [114].

Second, organizational consideration of occupational identity–related support environments may be relevant. Occupational identity may be related to how individuals interpret their work in connection with broader social value [47], and such identity-related resources may be associated with how work experiences are interpreted and with job involvement [48]. In this context, the findings suggest that occupational identity may be related to the conditions under which the association between psychological safety and PTSD symptoms may be more pronounced. This implies that understanding how organizational resources are perceived and utilized may require simultaneous consideration of individual identity-related resources [66].

Third, more structured approaches to peer support systems at the organizational level may be considered. Emotional support and shared experiences among peers have been discussed as relevant social resources in high-risk occupations [107,115,116]. Recent literature indicates that peer support is increasingly discussed as a promising approach in first responder populations, while also noting variability in definitions and implementation practices, suggesting the need for systematic design and evaluation [75]. Pilot studies with firefighters have reported that peer support training is related to self-efficacy and empathy-related skills [83], and implementation studies in public safety organizations have indicated that app-based peer support may be related to feasibility across diverse organizational contexts [117]. These findings may inform the development and future evaluation of organizational resource-based intervention approaches aimed at strengthening psychological safety and peer support in high-risk occupational settings.

### 4.4. Limitations and Future Research

Several limitations should be considered when interpreting the findings of this study.

First, this study was conducted using a cross-sectional design, which limits the ability to clarify the temporal ordering among variables [84]. Future research may benefit from longitudinal designs, including cohort-based approaches, to examine how associations among psychological safety, occupational identity, peer support, and PTSD symptoms develop over time.

Second, the sample was drawn from firefighters in a specific regional context, which requires cautious interpretation regarding generalizability [17]. Future studies may consider including more diverse samples across different regions or types of public safety personnel.

Third, this study relied on self-reported survey data, which may be subject to common method bias [100]. Although procedural and statistical approaches were applied, responses regarding psychological safety may have been influenced by social desirability bias and organizational response bias in a hierarchical occupational context. Future research may benefit from multi-source data, including objective mental health indicators or clinical assessments, and designs that better reflect operational environments in fire service settings [118].

Fourth, PTSD symptoms were assessed using an abbreviated eight-item version adapted from the PCL-5 rather than the full standard instrument. Although this approach was intended to capture core occupationally relevant symptom domains while reducing respondent burden, the modified measure may not fully reflect the breadth of the original construct and should be interpreted as a symptom indicator rather than a full-scale symptom assessment. Future research may examine whether the present findings are consistent when using the full PCL-5 or other comprehensive measurement approaches.

## 5. Conclusions

This study examined the association between psychological safety and PTSD symptoms among firefighters and investigated how this association is conditioned by occupational identity and peer support.

The findings indicated that psychological safety was negatively associated with PTSD symptoms, and that this association was conditioned by occupational identity and peer support. These findings suggest that organizational, individual, and social resources may be jointly related to PTSD symptom patterns in high-risk occupational contexts.

Within the limitations of a cross-sectional design, this study contributes to understanding how psychological safety, occupational identity, and peer support may be associated with mental health-related patterns among firefighters. These findings may inform understanding of occupational mental health in high-risk work environments and provide a basis for future research examining these associations in broader and longitudinal contexts.

## Figures and Tables

**Table 1:** Demographic characteristics of participants (N = 314).

Variable	Category	n	%
Gender	Male	281	89.5
	Female	33	10.5
Age	20s	7	2.2
	30s	105	33.4
	40s	93	29.6
	50s or older	109	34.7
Marital status	Single	47	15.0
	Married	267	85.0
Education	High school or less	53	16.9
	College enrolled	10	3.2
	College graduate	239	76.1
	Graduate degree	12	3.8
Position	Firefighter	10	3.2
	Senior Firefighter	79	25.2
	Fire Sergeant	67	21.3
	Fire Lieutenant	84	26.8
	Captains or higher	74	23.6
Years of service	Less than 5 years	26	8.3
	5–10 years	85	27.1
	10–20 years	88	28.0
	More than 20 years	115	36.6

Note. Percentages are based on valid responses.

**Table 2:** Measurement Model Fit Indices.

χ^2^	Df	*p*	CFI	TLI	RMSEA	90% CI (Lower–Upper)	SRMR
759.718	246	<0.001	0.915	0.905	0.082	0.075–0.088	0.048

**Table 3:** Reliability and convergent validity of the study constructs.

Construct	α	Ω	AVE
Psychological Safety	0.910	0.911	0.720
Post-Traumatic Stress	0.930	0.930	0.626
Occupational Identity	0.887	0.889	0.574
Peer Support	0.938	0.941	0.729

Note. α = Cronbach’s alpha; Ω = McDonald’s omega (composite reliability); AVE = average variance extracted. One psychological safety item (PS1) was excluded from the final measurement model due to a relatively low factor loading in the initial CFA.

**Table 4:** Descriptive statistics and correlations among study variables (N = 314).

Variable	M	SD	1	2	3	4
Psychological Safety	26.10	6.45	0.849			
Post-Traumatic Stress	22.97	11.54	−0.395 **	0.791		
Occupational Identity	28.32	6.69	0.393 **	−0.343 **	0.758	
Peer Support	32.86	6.79	0.639 **	−0.458 **	0.572 **	0.854

Note. M = mean; SD = standard deviation. Pearson correlations are presented below the diagonal. Values on the diagonal represent the square root of the average variance extracted (√AVE). ** *p* < 0.01.

**Table 5:** Association between psychological safety and post-traumatic stress.

Variable	B	SE	β	t	*p*
(Constant)	41.638	2.529		16.461	<0.001
Position (managerial = 1)	−0.212	1.555	0.008	0.137	0.891
Tenure (≥10 years = 1)	−0.031	1.380	−0.001	−0.023	0.982
Psychological Safety	−0.707	0.093	−0.395	−7.599	<0.001

Note. Dependent variable = post-traumatic stress. Position and tenure were included as control variables. B = unstandardized coefficient; SE = standard error; β = standardized coefficient.

**Table 6:** Moderated regression results (PROCESS Model 1).

Predictor	Model 2: (OI), B (SE)	Model 3: (PSU), B (SE)
Constant	41.502 (2.471) ***	40.911 (2.388) ***
Position (Managerial = 1)	−0.198 (1.542)	0.084 (1.555)
Tenure (≥10 years = 1)	−0.064 (1.366)	0.098 (1.380)
Psychological Safety (PS)	−0.356 (0.062) ***	−0.201 (0.072) **
Occupational Identity (OI)	−0.301 (0.071) ***	—
Peer Support (PSU)	—	−0.465 (0.083) ***
PS × OI	−0.109 (0.050) *	—
PS × PSU	—	−0.106 (0.047) *
R^2^	0.212	0.242
F	F(5, 308) = 16.550 ***	F(5, 308) = 19.623 ***

Note. B = unstandardized coefficient; SE = standard error; — = not included in the model. Continuous predictors were mean-centered. Position and tenure were included as control variables. * *p* < 0.05, ** *p* < 0.01, *** *p* < 0.001.

## Data Availability

The data presented in this study are available on reasonable request from the corresponding author. The data are not publicly available due to privacy and ethical restrictions.

## Supplementary Materials

The following supporting information can be downloaded at: https://www.mdpi.com/article/10.3390/ijerph23050635/s1, Table S1: Standardized factor loadings for the final measurement model; Table S2: Conditional effects of psychological safety on post-traumatic stress at levels of occupational identity; Table S3: Conditional effects of psychological safety on post-traumatic stress at levels of peer support; Table S4: Johnson–Neyman analysis results; Figure S1: Johnson–Neyman plot illustrating the conditional association between psychological safety and PTSD symptoms across levels of occupational identity. The shaded region indicates where this as-sociation is statistically significant; Figure S2: Johnson–Neyman plot illustrating the conditional association between psychological safety and PTSD symptoms across levels of peer support. The shaded region indicates where this association is statistically significant.
