# Supplementary material for: Association of Psychological Safety with PTSD Symptoms Among Regional Firefighters in South Korea: Moderating Roles of Occupational Identity and Peer Support
Source: Int J Environ Res Public Health. 2026 May 11;23(5):635. doi: 10.3390/ijerph23050635 (PMC13206716; doi:10.3390/ijerph23050635)
Supplement: Supplementary file 1 [file ijerph-23-00635-s001.zip › ijerph-4274018-supplementary.pdf]

Supplementary Materials

Table S1. Standardized factor loadings for the final measurement model.

Construct	Indicator	λ	Construct	Indicator	λ
Psychological Safety (PS)	PS2	0.827	Occupational Identity (OI)	OI1	0.788
	PS3	0.842		OI2	0.844
	PS4	0.856		OI3	0.774
	PS5	0.870		OI4	0.877
Post-Traumatic Stress (PTSD)	PTSD1	0.750		OI5	0.611
	PTSD2	0.736		OI6	0.716
	PTSD3	0.687	Peer Support (PSU)	PSU1	0.790
	PTSD4	0.781		PSU2	0.830
	PTSD5	0.790		PSU3	0.877
	PTSD6	0.863		PSU4	0.834
	PTSD7	0.867		PSU5	0.873
	PTSD8	0.872		PSU6	0.893

Note. All standardized factor loadings were statistically significant ($p < 0.001$).

Table S2. Conditional effects of psychological safety on post-traumatic stress at levels of occupational identity.

Level of Occupational Identity	Effect (B)	SE	t	95% CI		p
				LL	UL	
−1SD (Low)	−0.234	0.079	−2.953	−0.390	−0.078	0.003
Mean	−0.356	0.062	−5.777	−0.477	−0.235	0.001
+1SD (High)	−0.478	0.086	−5.538	−0.648	−0.307	0.001

Note. Conditional effects represent the association between psychological safety and PTSD symptoms at low (−1 SD), mean, and high (+1 SD) levels of occupational identity.

Table S3. Conditional effects of psychological safety on post-traumatic stress at levels of peer support.

Level of Peer Support	Effect (B)	SE	t	95% CI		p
				LL	UL	
−1SD (Low)	−0.080	0.087	−0.922	−0.252	0.092	0.357
Mean	−0.201	0.072	−2.783	−0.342	−0.059	0.006
+1SD (High)	−0.321	0.091	−3.519	−0.500	−0.141	0.001

Note. Conditional effects represent the association between psychological safety and PTSD symptoms at low (−1 SD), mean, and high (+1 SD) levels of peer support.

Table S4. Johnson–Neyman analysis results.

Moderator	J–N Threshold (SD)	Interpretation
Occupational Identity	−1.553	The association between psychological safety and PTSD symptoms becomes statistically significant when occupational identity exceeds this value.
Peer Support	−0.504	The association between psychological safety and PTSD symptoms becomes statistically significant when peer support exceeds this value.

Note. The Johnson–Neyman technique was used to identify the region of significance ($p < 0.05$).

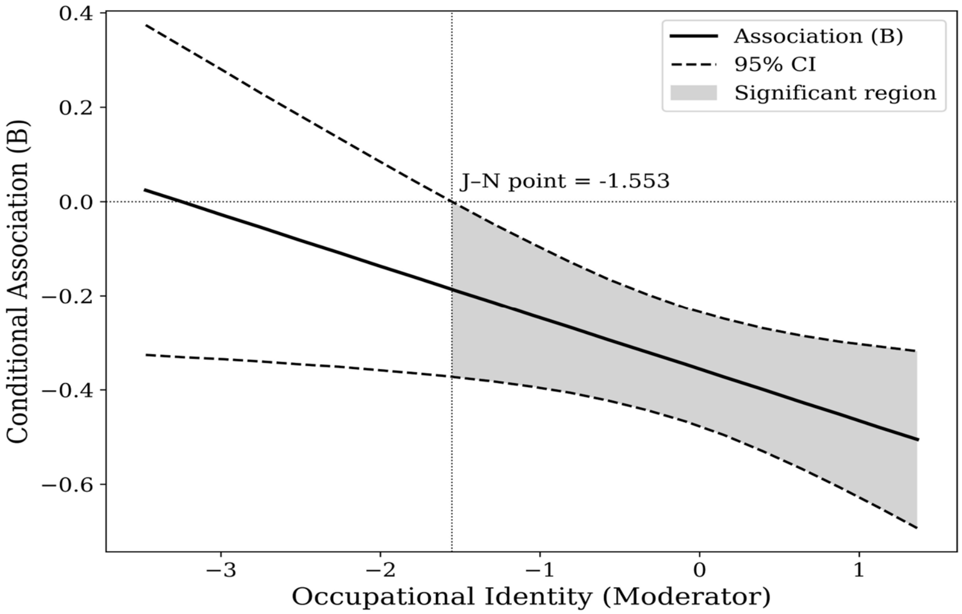

Figure S1. Johnson–Neyman plot illustrating the conditional association between psychological safety and PTSD symptoms across levels of occupational identity. The shaded region indicates where this association is statistically significant.

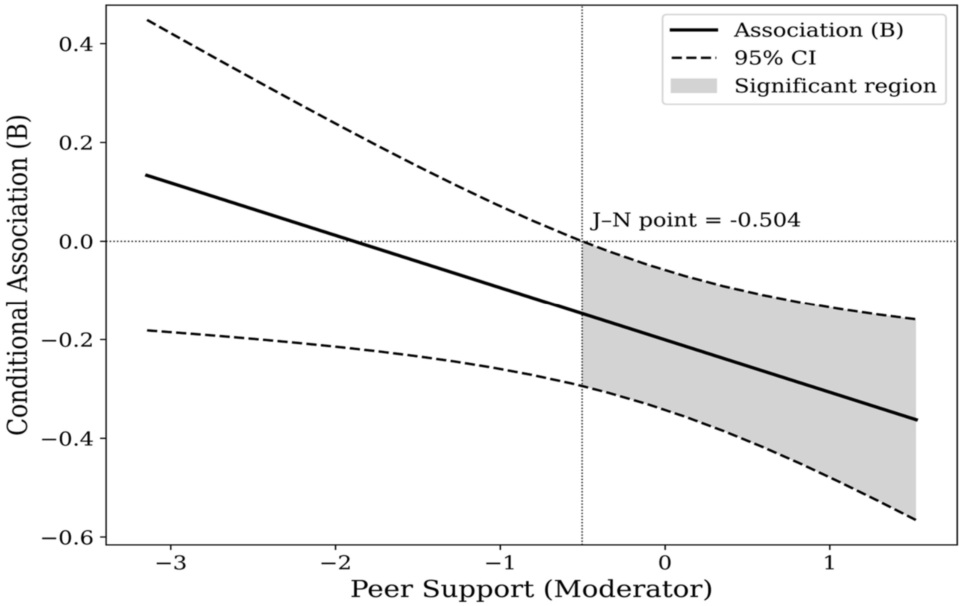

Figure S2. Johnson–Neyman plot illustrating the conditional association between psychological safety and PTSD symptoms across levels of peer support. The shaded region indicates where this association is statistically significant.
